# Supplementary material for: Learning curve and surgical outcome of robotic assisted colorectal surgery with ERAS program
Source: Sci Rep. 2022 Nov 29;12:20566. doi: 10.1038/s41598-022-24665-w (PMC9709162; doi:10.1038/s41598-022-24665-w)
Supplement: Supplementary file 1 — Supplementary Information 1. [file 41598_2022_24665_MOESM1_ESM.zip › LSC Raw data-Ñ[▒K/118/N_ñw╝╨░O▒Kñσ.pdf]

W55 / W55

臺中榮民總醫院

手術室護理記錄

[redacted]

基本資料

- 室別： 二醫手術室-31室，手術科別： CRS，手術類別： 常規，日期： 2021/02/25
- 進入時間： 1200 ； 麻醉開始時間： 1200 ； Time Out時間： 1320 ； 劃刀時間： 1321 ；
- 麻醉方式： GE

  診斷： Malignant tumor of sigmoid colon

  手術名稱： Laparoscopic Anterior resection and anastomosis - malignant

  手術局部用藥： nil

  術前預防性抗生素：
  - 抗生素名稱一： Veterin 1GM IVD/AR
  - 時間： 1240
  - 上次簽章: 李庭

  ，追加預防性抗生素：
- 手術醫師： 蔣鋒帆
- 麻醉醫師： VS 施涵薰
-
  - 刷手護士A ： 李庭，時間： 1200，接替刷手一： 顏育銹，時間： 1230，接替刷手二： 李庭，時間： 1500
  - 流動護士A ： 許育庭，時間： 1200，接替流動一： 李庭，時間： 1300，接替流動二： 王心俞，時間： 1500
- 更多接替及流動護士： 接替刷手三： 許育庭，時間： 1800，接替流動三： 李宜芳，時間： 1730，接替流動四： 王心俞，時間： 1800

皮膚狀況

A-V Shunt： 無

術前： 不完整

- 2.瘀傷

部位(術前)：

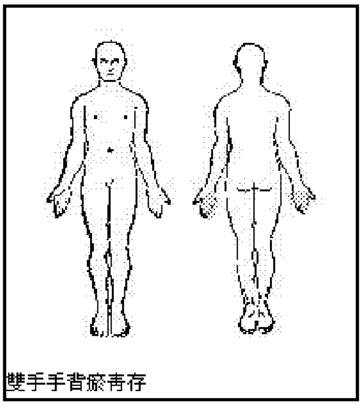

記錄者： 上次簽章: 許育庭

術後： 不完整

- 2.瘀傷

部位(術後)：

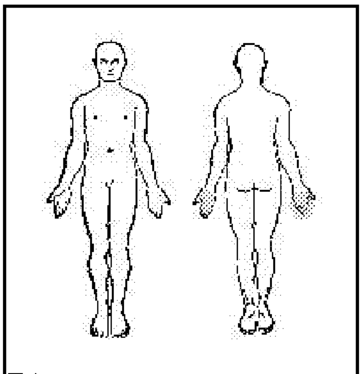

記錄者： 上次簽章: 李庭

手術準備

手術臥式 單一手術臥式

-
    - 手術臥式： 截石臥式
    - 使用附件： 溫毯 38 ℃，矽膠枕頭，腳架，其它： 頭架、左手架
    - 皮膚準備： 10%Aq Betadine，Hibiscrub，2% Chlorhexidine
    - 電燒： 使用電燒: 電極片位置： 其它： 背部迴路電極板，記錄者： 上次簽章: 許育庭

止血帶T： 無

植入物

- 植入物名稱/號碼/數量/部位： SURGIWRAP/0.02*100*130mm/數量壹/骨盆腔

, 記錄者： 王心俞

標本送檢

- 細菌培養 無
- 細胞學 無
- 冰凍切片 無
- 病理標本
  組織名稱一： D-colon
  ,
  組織名稱二： IMA root
  ,
  組織名稱三： distal cut end
- 截肢 無

標本交付 標本： 3 袋，標本： 0 盒，標本： 0 瓶，細胞學： 0 管，Culture Tube： 0 管，被交付醫師： R 林志安，

- 記錄者： 王心俞
- 時間： 1630

傷口術後敷料

傷口敷料： 傷口敷料： 無

- 請於圖上標示部位：

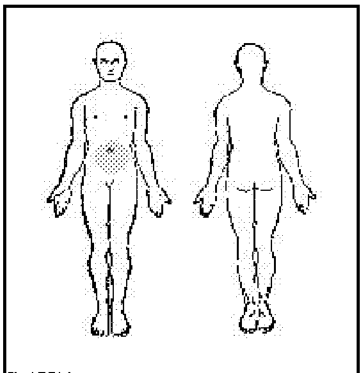

- 傷口敷料： mepore

導管

導管	天數	導管資訊	插入日期	插入時間	更換日期	拔除日期	拔除時間	拔除原因	預計拔除/更換	預計拔除/更換日期
Foley(1)	1	號碼:16 Fr.; 部位:膀胱;	2021/02/25	1235						
Penrose Drain(1)	1	號碼:12 mm. mm.; 部位:骨盆腔; 註記:左腹;	2021/02/25	1830						
A-line (1)	1	號碼:22 號; 部位:左手;	2021/02/25	1220						

用物計數

- 手術中出血量： 200 c.c.
- 手術中尿量： 600 c.c.
-
    - 其它： 無

用物計數： 多切口同時縫合

- 1.器械： 正確，2.縫針： 正確，3.刀片： 正確，4.紗布： 正確，5.腹部墊： 未使用，6.紗球： 未使用，7.PEANUTS ： 未使用，8.紗條： 未使用，9.絲帶： 未使用，10.針頭： 未使用，11.Vessel loop： 未使用，12.Boots： 未使用，13.Jelco： 未使用，14.棉條： 未使用，
    - 第一次：
        - 刷手護士 ： 許育庭
        - 流動護士： 王心俞
        - 時間： 1900

  ,
    - 第二次：
        - 刷手護士 ： 許育庭
        - 流動護士： 王心俞
        - 時間： 1920

  ,
    - 第三次：
        - 刷手護士 ： 許育庭
        - 流動護士： 王心俞
        - 時間： 1930

備註

備註：NIL

- 記錄者： 王心俞 上次簽章: 許育庭
- 時間： 1945

填寫護理人員 王心俞

正式摘要

W55 / W55

doctorRank:B
